# Chromosomal Instability Estimation Based on Next Generation Sequencing and Single Cell Genome Wide Copy Number Variation Analysis

**DOI:** 10.1371/journal.pone.0165089

**Published:** 2016-11-16

**Authors:** Stephanie B. Greene, Angel E. Dago, Laura J. Leitz, Yipeng Wang, Jerry Lee, Shannon L. Werner, Steven Gendreau, Premal Patel, Shidong Jia, Liangxuan Zhang, Eric K. Tucker, Michael Malchiodi, Ryon P. Graf, Ryan Dittamore, Dena Marrinucci, Mark Landers

**Affiliations:** 1 Epic Sciences, Inc., San Diego, CA, United States of America; 2 Genentech, Inc./ Roche, San Francisco, CA, United States of America; King's College London, UNITED KINGDOM

## Abstract

Genomic instability is a hallmark of cancer often associated with poor patient outcome and resistance to targeted therapy. Assessment of genomic instability in bulk tumor or biopsy can be complicated due to sample availability, surrounding tissue contamination, or tumor heterogeneity. The Epic Sciences circulating tumor cell (CTC) platform utilizes a non-enrichment based approach for the detection and characterization of rare tumor cells in clinical blood samples. Genomic profiling of individual CTCs could provide a portrait of cancer heterogeneity, identify clonal and sub-clonal drivers, and monitor disease progression. To that end, we developed a single cell Copy Number Variation (CNV) Assay to evaluate genomic instability and CNVs in patient CTCs. For proof of concept, prostate cancer cell lines, LNCaP, PC3 and VCaP, were spiked into healthy donor blood to create mock patient-like samples for downstream single cell genomic analysis. In addition, samples from seven metastatic castration resistant prostate cancer (mCRPC) patients were included to evaluate clinical feasibility. CTCs were enumerated and characterized using the Epic Sciences CTC Platform. Identified single CTCs were recovered, whole genome amplified, and sequenced using an Illumina NextSeq 500. CTCs were then analyzed for genome-wide copy number variations, followed by genomic instability analyses. Large-scale state transitions (LSTs) were measured as surrogates of genomic instability. Genomic instability scores were determined reproducibly for LNCaP, PC3, and VCaP, and were higher than white blood cell (WBC) controls from healthy donors. A wide range of LST scores were observed within and among the seven mCRPC patient samples. On the gene level, loss of the *PTEN* tumor suppressor was observed in PC3 and 5/7 (71%) patients. Amplification of the androgen receptor (*AR*) gene was observed in VCaP cells and 5/7 (71%) mCRPC patients. Using an *in silico* down-sampling approach, we determined that DNA copy number and genomic instability can be detected with as few as 350K sequencing reads. The data shown here demonstrate the feasibility of detecting genomic instabilities at the single cell level using the Epic Sciences CTC Platform. Understanding CTC heterogeneity has great potential for patient stratification prior to treatment with targeted therapies and for monitoring disease evolution during treatment.

## Introduction

Cancer is a genetic disease. The accumulation of genetic and epigenetic lesions in response to environmental exposures to carcinogens and/or random cellular events often results in the inactivation of tumor suppressor genes that play critical roles in the maintenance of cell cycle, DNA replication and DNA repair [1,2]. Loss or inhibition of cellular DNA repair mechanisms often results in an increased mutation burden and genomic instability. Genomic instability is an important driver of sub-clonal heterogeneity and is frequently observed in solid tumors between different lesions [3,4], within the same tumor [5,6], and even within the same solid biopsy site [7–9]. The resulting increase in tumor cell heterogeneity and the presence of multiple sub-clonal driver alterations complicate therapeutic intervention with targeted therapies aimed at inhibiting a single molecular target [5,6].

Copy number variations (CNV) are prevalent across many cancer types [2]. The corresponding gain of oncogenes and/or loss of tumor suppressors are frequent drivers of disease progression, and are correlated with therapeutic response or resistance [10,11]. For example, *PTEN* loss is frequent in many tumor types and is associated with sensitivity to PI3K inhibitors [12], whereas human epidermal growth receptor 2 (*HER2*) amplification in breast cancer is tested for guiding HER2 targeted therapy [13]. In addition to the identification of focal CNV driver alterations, genome-wide CNV profiles can be used to characterize genomic instability [14,15], often associated with therapeutic response. Increased frequency of large-scale state transitions (LST) genome wide (the number of CNV breakpoints >10Mb) and LOH, have been associated with therapeutic response for both platinum-based chemotherapy and PARP inhibitors [16–18].

Measurement of copy number alterations is currently most assessable via surgical specimen or biopsy, although not without complication. The biopsy process can be invasive and prone to sampling error due to intra-tumor heterogeneity and the state of disease progression [19,20], and may not reflect the dynamic and heterogeneous tumor cell population. As a result, liquid biopsy approaches have been developed to understand disease progression in real time from a non-invasive blood draw, through analysis of either cell-free DNA (cfDNA) or circulating tumor cells (CTCs) that are present in patients’ blood [21–24].

The detection of focal CNV events from isolated cfDNA samples is possible [11]; however, this method can only detect gene amplifications or genetic mutations. Detection of tumor suppressor loss and genomic instability from cfDNA are complicated by the relatively small amount of tumor-derived DNA in cfDNA, by the lack of whole genome coverage and by the presence of CNV alterations at sub-clonal levels [25].

Recent studies have demonstrated that circulating tumor cells (CTCs) reflect tumor heterogeneity and represent the active metastatic population more accurately than archival tumor biopsy [26]. While other CTC detection methods rely on positive selection based on cell surface marker expression, or enrichment of CTCs based on size, morphology, or depletion of CD45+ cells, the Epic Sciences CTC Platform utilizes a non-enrichment approach to detect Cytokeratin positive (CK)+, CK-, cluster, and apoptotic CTCs [27–29], and is well-suited for characterizing heterogeneous sub-clonal populations of CTCs in metastatic disease. In this study we analyzed both traditional CD45-/CK+ CTCs and CD45-/CK- CTCs. Previous studies have shown that DNA from individual CTCs can be whole genome amplified and sequenced to identify loss of tumor suppressors or gain of oncogenes, and to identify sub-clonal populations of CTCs within heterogeneous populations [8,10,30,31].

Building on this work, we developed a reproducible genomic assay integrated with the Epic Sciences CTC Platform to further characterize CTCs for the presence of sub-clonal tumor suppressor loss, oncogene amplification and genomic instability with single cell resolution. Here, we present an analytical validation of a single-CTC CNV assay on the Epic Sciences CTC Platform and demonstrate its feasibility and clinical relevance in a small cohort of mCRPC patient samples. The assay demonstrates the technical capability to expand our understanding of sub-clonal evolution in cancer and can be used to further elucidate the role of genomic instability as a mechanism of resistance to therapy.

## Materials and Methods

### Sample Processing

For cell line controls, healthy donor blood was collected in Cell-free DNA BCT™ (Streck) tubes, spiked with cell line cells, and the slides were prepared and stained as previously described [27]. For patient samples, peripheral blood samples from seven (7) patients with metastatic castration resistant prostate cancer (mCRPC) were collected in CellSave tubes (Janssen Diagnostics) and shipped to Epic Sciences (San Diego, CA) at ambient temperature, and processed onto slides as previously described [26,27]. Briefly, red blood cells were lysed, and all nucleated cells were deposited onto glass microscopy slides at a density of 3 x 10^6^ cells/slide and stored at -80°C prior to staining. Three cell lines representative of prostate cancer with known genetic changes were used: VCaP (ATCC catalog #CRL-2876), LNCaP (ATCC catalog #CRL-1740), and PC3 (ATCC catalog #CRL-1435).

### CTC Enumeration

Slides were stained with a cocktail of CK antibodies to identify CTCs, CD45 to detect white blood cells (WBCs), DAPI to stain cell nuclei, and Androgen Receptor (AR) antibody to detect AR protein. Stained slides were scanned using Epic Sciences’ rapid fluorescent scanning method and analyzed using a multi-parametric digital pathology algorithm to detect CK^+^/AR^+/-^/CD45^-^/DAPI^+^ CTC candidates. For patient samples, 2 slides per sample were tested for CTC enumeration and N-terminal AR expression as described previously [27]. Classification of the CTC candidates identified by the algorithm was confirmed by trained technicians. After staining, slides were stored at -80°C before further processing.

### Cell Isolation, Amplification, and Next-Generation Sequencing

Individual CTCs were identified by the Epic Sciences’ rapid fluorescent scanning method and X/Y coordinates for each CTC were recorded. Non-apoptotic CTCs were relocated on the slide using an inverted fluorescent microscope, and single cells were individually isolated into PCR tubes using an Eppendorf TransferMan NK4 micromanipulator. For cell line controls, multiple single cell replicates (8 LNCaP, 8 PC3, 5 VCaP, and 4 WBCs) were processed from each cell line and WBCs. For patient CNV analysis, 67 individual CTCs from the 7 mCRPC samples were sequenced. A subset of patient CTCs with diverse phenotypes were selected for sequencing based on nuclear size, CK expression, and N-term AR expression. Single cells were lysed in 1.5 μL of high pH lysis buffer as described previously [10]. Single cell whole genome amplification (WGA) was performed using SeqPlex Enhanced (Sigma), and NGS libraries were constructed with NEBNext Ultra DNA Library Prep Kit for Illumina (NEB) using 100 ng of WGA DNA, as per manufacturers’ recommendations, with minor modifications. Library size distributions were analyzed on a Fragment Analyzer (Advanced Analytical), and library concentrations were determined by real-time PCR (NEBNext Library Quant Kit for Illumina, NEB). Libraries were pooled in equinanomolar ratios and sequenced on an Illumina NextSeq 500 using a High Output kit in a Paired-End 2x150 format (PE 2x150).

### PTEN FISH

*PTEN* FISH was performed on separate slides containing at least 3 or more enumerated CTCs, as described previously [26]. Briefly, a Cymogen Dx 2-color probe specific to *PTEN* and chromosome 10 centromeres (CEP10) was used, and slides were counterstained with DAPI. As an internal control on every slide, 20 WBCs were evaluated for *PTEN*, and each CTC was classified for *PTEN* loss according to the number of FISH signals: *PTEN* homozygous (HO) loss (PTEN = 0 and CEP10 ≥ 1), Hemizygous (HE) loss (PTEN = 1 and CEP10 ≥ 1), or Non-Deleted (PTEN ≥ 2 and CEP10 ≥ 1), and a *PTEN* call was made for each sample based upon the scoring algorithm as previously described [26].

### Patient Samples

Blood samples were collected at screening from patients with histologically confirmed mCRPC with a treatment history of docetaxel-based chemotherapy (including docetaxel and/or cabazitaxel), and who had progressed during treatment with at least one hormonal therapy (luteinizing hormone-releasing hormone, bicalutamide, etc.), and showed radiographic evidence of disease progression or showed two rising PSA levels that meet the Prostate Cancer Working Group 2 (PCWG2) consensus criteria [32] prior to enrollment in the clinical trial (NCT01485861). The study was conducted in accordance with Good Clinical Practice guidelines and the Declaration of Helsinki. Patients from the following sites participated in this study: Barbara Ann Karmanos Cancer Institute, Sarah Cannon Research Institute, and Pacific Hematology Oncology Associates. Studies were approved by the Institutional Review Boards (IRB) at Karmanos Cancer Institute, Sarah Cannon Research Institute, and Pacific Hematology Oncology Associates. Written informed consent was obtained from all patients before enrollment, in agreement with approved protocols from respective ethics committees at every site. Patient CTC counts and metrics are summarized in Table 1.

**Table 1:** Patient demographics.

Patient ID	CTCs/mL	Total CTC Population			Number CTCs sequenced	CTCs Sequenced		
		Nuclear Area μm^2^range (mean ± sd)	CK IF	% AR N-Term IF Positive		Nuclear Area μm^2^ range (mean ± sd)	CK IF	% AR N-Term IF Positive
**1**	121.9	26.81–224.76 (59.53±26.46)	100% CK^+^, 0% CK^-^	3%	9	34.02–79.38 (56.25±14.94)	100% CK^+^, 0% CK^-^	22%
**2**	114.5	41.24–248.41 (84.08±35.81)	80.3% CK^+^, 19.7% CK^-^	76%	16	43.3–248.47 (100.15±54.1)	78.6% CK^+^, 21.4% CK^-^	57%
**3**	45.5	16.5–217.54 (76.71±40.9)	76.9% CK^+^, 23.1% CK^-^	58%	17	30.93–217.54 (76.36±58.1)	100% CK^+^, 0% CK^-^	47%
**4**	4.8	45.36–53.61 (49.83±4.17)	100% CK^+^, 0% CK^-^	0%	2	45.36–53.61 (49.83±4.17)	100% CK^+^, 0% CK^-^	0%
**5**	4.3	39.18–139.19 (69.59±47.1)	100% CK^+^, 0% CK^-^	0%	3	39.18–57.74 (46.4±9.94)	100% CK^+^, 0% CK^-^	0%
**6**	8.8	30.93–151.56 (64.78±44.6)	100% CK^+^, 0% CK^-^	33%	5	30.93–151.56 (68.93±41.66)	100% CK^+^, 0% CK^-^	40%
**7**	70.8	17.53–178.36 (69.49±31.88)	91.4% CK^+^; 8.6% CK^-^	63%	15	38.15–178.36 (71.56±35.1)	80% CK^+^, 20% CK^-^	60%

Abbreviations: Androgen Receptor, AR; Cytokeratin, CK; Circulating Tumor Cell, CTC; Immunofluorescence, IF; standard deviation, sd

### Data Analysis

**Fig 1:**
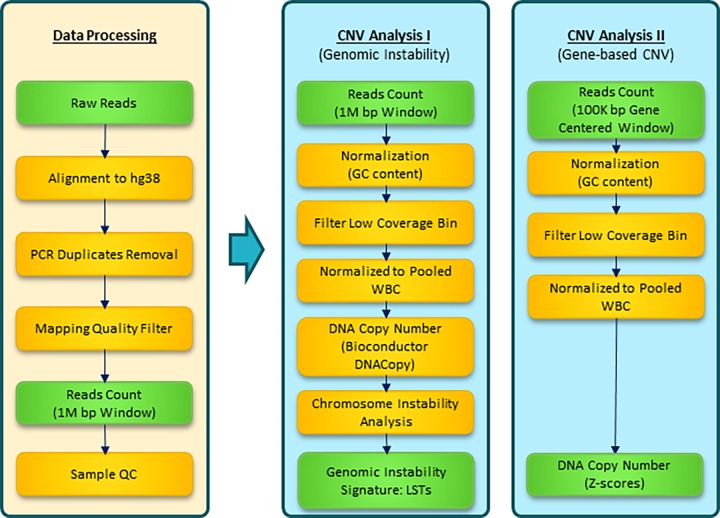
Epic Sciences single cell NGS-based CNV analysis pipeline. FASTQ files were aligned to hg38 human reference genome from UCSC using BWA. BAM files with a MAPQ quality score greater than 30 were kept for further analysis with two separate pipelines: analysis pipeline 1 for genomic instabilities estimation using 1M bp window; analysis pipeline 2 for determining copy number alterations of individual genes.

Burrows-Wheeler Aligner (BWA, http://bio-bwa.sourceforge.net) was used to map FASTQ files to the Hg38 human reference genome (UCSC, http://hgdownload.soe.ucsc.edu/goldenPath/hg38/bigZips/) and alignment (BAM) files were generated. BAM files were filtered for quality using MAPQ30 as a cutoff and PCR duplicate reads were also removed. The filtered alignment files were further processed using two separate pipelines (Fig 1). To generate a CNV analysis control genome from single cell WGA DNA, 15 WBCs were collected from different human adult male individuals without hematological disease and were used as a universal reference. For each sample, read counts per bin (window size per bin varies between two pipelines, see below) were normalized proportionally to bring the total read counts to 1 million. Then median, mean, and standard deviation (sd) of normalized reads number of these controls were calculated for each bin for further use.

Analysis pipeline 1 was utilized for genomic instabilities estimation. Approximately 3000 bins of 1 million base pairs each were generated from the Hg38 human reference genome. After sequencing, reads were counted within each bin for each sample. For each sample, read counts per bin were normalized proportionally to make the total read counts to 1 million, followed by GC content adjustment for each bin. Sample codes for this analysis have been published previously [33,34]. Median values of each bin read counts of WBC controls were used to exclude low coverage bins from downstream analyses (<100 reads). Ratios between test samples and WBC controls were calculated and reported after Log2 transformation. Chromosomal segments were predicted using R Bioconductor package DNAcopy (alpha = 0.05), see the sample code from the literature [33,34] which determined break points where DNA copy number changed. LSTs were calculated as number of chromosomal breaks between adjacent regions of at least 10 Mb. Patterns of whole genome copy number variations were visualized with Circos [35].

Analysis pipeline 2 was used to calculated copy number alterations for individual genes. RefSeq gene hg38 coordinates were downloaded from UCSC genome browser. To improve the specificity of copy number calling for shorter genes (< 100K bp between the start and end position of the transcript on the genome), the gene coordinates were equally extended at both 5’ and 3’ ends of locus the to have a final span of 100K bp. Reads were counted within each gene’s final coordinates, and the counts were normalized to 1 million total and to GC content. Low coverage bins (<10 reads in WBC controls) were removed from downstream analysis. Ratios between test samples and WBC controls were calculated and reported after Log2 transformation. Z scores were calculated for each gene by comparing the test sample and WBC controls, $Z=\frac{X-\bar{X}}{S}$, where *X* is the normalized copy number of the test sample, $\bar{X}$ is the average copy number from the universal WBC reference, and *S* is the standard deviation from the universal reference. The single cell level cut-off for gene amplification is Z score >3; for deletion it is Z score <-3 [36]. At the patient level, amplification or deletion of a single gene needed to be observed on at least two CTCs for classification as alteration.

One BAM file from a single cell with the highest number of reads from each cell line (LNCaP, PC3, and VCaP), was randomly subsampled with SAMtools (e.g. samtools view -s 0.50). New BAM files were generated with 50%, 25%, 10% randomly selected reads. Genomic instabilities were determined on the subsampled BAM files to estimate the minimal reads requirement.

Post-sequencing data Quality Control (QC) criteria was developed to measure data quality and library complexity. The criteria included minimum sequencing reads (>350K) and residual over reads ratio (>1.9), which was calculated as the ratio of residuals of LOESS fitting (Bioconductor LOESS function) over total sequencing reads.

## Results

### Cell Line Single CTC Identification, Isolation, WGA, and Sequencing

**Fig 2:**
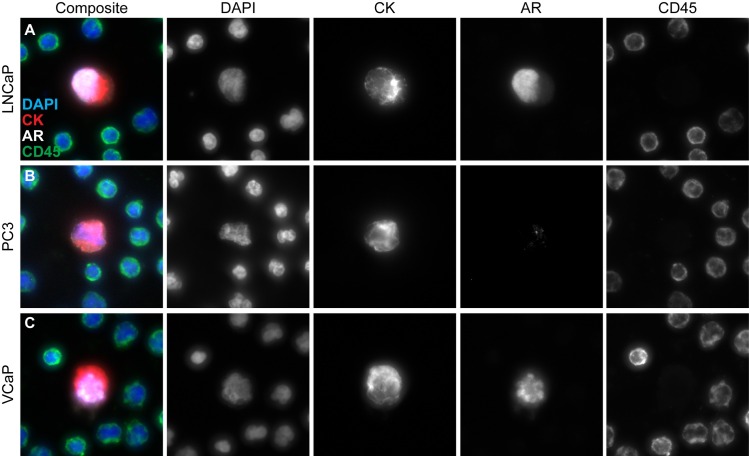
Representative images of CTCs identified. Representative CTC images from cell lines (A) LNCaP, (B) PC3, and (C) VCaP. Slides were stained with CK, AR, CD45, and DAPI. Individual CTCs were identified by Epic Sciences’ algorithm and visually confirmed.

LNCaP, PC3, and VCaP cell lines were selected as representative prostate cancer cell lines with known genomic aberrations. LNCaP cells are characterized by a heterozygous *PTEN* deletion, VCaP cells harbor an amplification of the androgen receptor (*AR*) on the X chromosome, and PC3 cells have a homozygous *PTEN* deletion [37]. Cells were imaged using 4 channels: DAPI, CK, AR, and CD45. The presence of CK expression and lack of CD45 expression combined with intact DAPI meets the standard definition of CTC and is consistent with their epithelial origin [23,38]. The lack of AR protein in PC3 cells, despite an intact *AR* gene, is consistent with previously published studies [37,39]. The very high levels of AR protein in VCaP cells is consistent with a previously published AR gene amplification [40]. Representative immunofluorescent images from LNCaP, PC3, and VCaP cell lines stained with CK, AR, CD45, and DAPI are shown (Fig 2A–2C).

**Fig 3:**
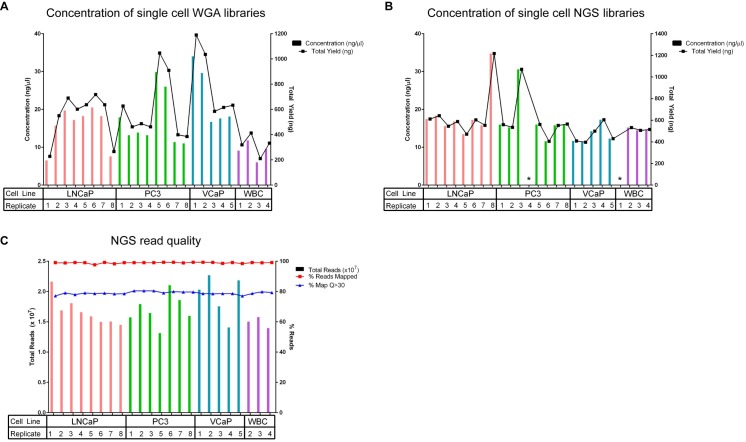
Whole Genome Amplification, library preparation quality control, read quality, output, and alignment quality. (A) DNA concentrations and total yield for each single cell WGA as measured by UV/Vis. Libraries were constructed from independent replicates of single cells: 8 from LNCaP, 8 from PC3, 5 from VCaP, and 4 from WBCs. Overall, we achieved an average 100% success rate during the single cell whole genome amplification procedure: 8/8 single cells (100%) successfully amplified for PC3 and LNCaP cancer cell lines, while 5/5 (100%) single VCaP cells and 4/4 (100%) single WBCs amplified. An average yield of 578 ng (range of 227–1190 ng) was obtained from the single cell WGA reactions. (B) Concentrations of the next-generation sequencing libraries as measured by PicoGreen. All of the NGS libraries passed QC with adequate yield except for two samples that failed to render any detectable amount of library DNA product (one PC3 and WBC replica samples) (23/25; 92% success rate). An average yield of 581 ng (range of 397–1216 ng) was obtained among the single cell NGS libraries. (C) Assessment of NGS read quality. >99% of the NGS reads had an average PHRED score greater than Q30 pre-alignment, indicating high quality reads. An average of 17 million reads/sample (in each direction) were obtained. 99% of the reads mapped to the reference genome (hg38) with 79% of the reads mapping with a MAPQ score greater than 30. *Failed library preparation.

Eight LNCaP cells, 8 PC3 cells, 5 VCaP cells, and 4 WBCs were individually isolated for WGA and sequencing library preparation. The mean WGA yield was 578 ng (*n* = 25, with a range of 227–1190 ng) (Fig 3A). The mean library yield was 581 ng (*n* = 23, with a range of 397–1216 ng) (Fig 3B). While 100% of the single cells isolated had sufficient WGA DNA concentrations, 23/25 (92%) of the NGS libraries passed QC with adequate yield and were further processed for sequencing. An average of 17 million reads/sample (*n* = 23, with a range of 13–22 million) were obtained. 99% of the reads mapped to the reference genome (hg38) with 79% of the reads mapping with a MAPQ score greater than 30 (Fig 3C).

### Single Cell Sequencing Reproducibility in Cell Lines

**Fig 4:**
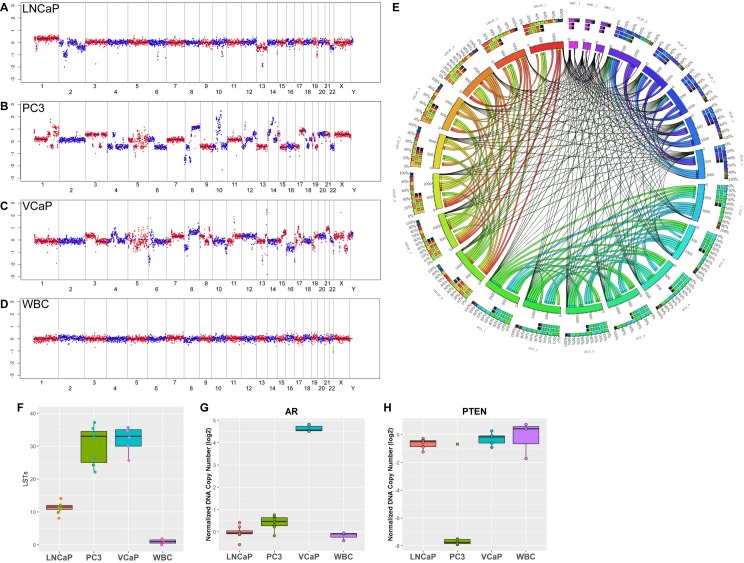
Prostate cancer cell line single cell CNV profiles, genomic instability scores and AR, PTEN copy number status. Whole genome copy number plots from prostate cancer cell lines (A) LNCaP, (B) PC3, and (C) VCaP, and (D) WBC controls. (E) Absolute Pearson correlation values (0–100%) were calculated across samples and viewed using Circos Table Viewer (http://circos.ca/presentations/articles/vis_tables1/). For visualization purposes, the top 25% highest correlations are displayed. Each color-coded segment represents a cell line replicate. Correlations between replicates are denoted by links or ribbons, the width of which is proportional to the degree of correlation. Much higher correlations were observed in intra-cell line comparisons than inter-cell line comparisons, indicating that the assay has good reproducibility regardless of cell line used. (F) Box-whisker plot of LST scores for prostate cancer cell lines and WBCs. All 3 cell lines had high LST scores compared to the WBCs, with PC3 and VCaP having the highest scores. (G) Box-whisker plot of log2 normalized DNA copy number in *AR*. Amplification of the *AR* gene was observed in the VCaP single cells reproducibly (5/5, 100%). This amplification was not observed in PC3 (0/7), LNCaP (0/8), or WBC controls (0/3). (H) Box-whisker plot of log2 normalized DNA copy number in *PTEN*. The VCaP cell line has non-deleted *PTEN* (0/5, 0%), while *PTEN* loss was detected in PC3 (6/7, 86%), LNCaP (1/8, 13%), and 1/3 WBC controls (1/3, 33%).

Whole genome CNV profiles from single cells of LNCaP, PC3, VCaP and WBCs were log2 normalized to visualize areas of amplification or deletion. The copy number profiles from each of the independent biological replicates from LNCaP, PC3, and VCaP cell lines (S1A–S1C Fig) demonstrate the reproducibility of the assay in that consistent CNVs are detected in all biological replicates within a cell line. Representative copy number profiles from each cell line and the WBC control are shown in Fig 4A–4D. Correlation coefficients across replicates of LNCaP, PC3, VCaP and WBCs were used to estimate the copy number analysis reproducibility (Fig 4E). Absolute Pearson correlation values from 0–100% within and between cell lines are represented as a circular diagram using Circos Table Viewer (http://mkweb.bcgsc.ca/tableviewer/). Each segment is color coded, denoting a cell line replicate. Links connecting each segment are represented as ribbons, the width of which corresponds proportionally to the degree of correlation. Intra-cell line replicates show thick ribbons connecting with each other, whereas inter-cell lines show thin ribbons, indicating that the assay is highly reproducible within each cell line. To further assess the reproducibility of our single cell sequencing, we analyzed single cells, pools of 5 cells, and pools of 10 cells, in replicates of 5 each for the LNCaP, PC3 and VCaP cell lines. Cells were pooled prior to WGA and analyzed by NGS. Replicate samples were combined for further analysis by calculating the median value of normalized copy number for each gene. The Pearson correlation coefficients were calculated for every pair of samples. The correlation analysis indicated that regardless of the number of cells sequenced, CNV profiles for all replicates correlated highly within each cell line, but not across cell lines (S1 Table). These data also indicate that copy number variations that were observed for each cell line were reproducible by our NGS methods, supporting our method of determining CNVs and genomic instability in a single cell. Furthermore, to determine the rate of false positives called by our assay, we analyzed the incidence of private CNV events as calculated by concordance analysis. The incidence of private CNV events, which are CNV events found within a single cell replicate that are not present in other cells within the same cell line, was low within cell lines (S1 Table). These analyses indicate that there is high rate of intra-cell line concordance due to the low number of false CNV events called.

### Cell Line Genomic Profiles and Instability Analysis

LST scores in the three tested prostate cancer cell lines and WBCs were determined (Fig 4F, Table 2). Significantly higher genomic instability signature scores were determined reproducibly for LNCaP, PC3, and VCaP, compared to WBC controls. These data are summarized in Table 2. Given the high mutation rates of these cell lines analyzed in bulk [41,42], our single cell LST analysis recapitulates these findings.

**Table 2:** Prostate Cancer Cell Line Genomic Instability Scores.

Cell Line	LST			
	mean	sd	p*	Coefficient Variation**
LNCaP (n = 8)	11.3	1.8	<0.001	15.6%
PC3(n = 7)	30.1	6.0	0.001	19.9%
VCaP(n = 5)	32.0	4.1	0.01	12.7%
WBC(n = 3)	1.0	1.0	N/A	100%

*p value is determined using Student’s t-test

**Coefficient of variation is defined as the ratio of the standard deviation σ to the mean μ

Abbreviations: Large Scale Transition, LST; standard deviation, sd; White Blood Cell, WBC

### Cell Line CNV Reproducibility

The normalized AR DNA copy number change on chromosome X corresponding to the *AR* gene is consistent with a previously published *AR* gene amplification in VCaP cells [40] (Fig 4G) and with the high expression of the AR protein (Fig 2C). By CNV analysis, *AR* gene amplification was observed in 5/5 VCaP cells, but not in any other cell line analyzed or the WBC controls (Fig 4G).

The normalized *PTEN* DNA copy number change on chromosome 10 corresponding to the *PTEN* gene is consistent with the known *PTEN* status in these cell lines: null in PC3 and reduced in heterozygous LNCaP cells but 2 copies in VCaP cells [43–45] (Fig 4H). Based on our CNV cutoff (Z score > 3 for amplification, or < -3 for loss), 1/8 LNCaP, 6/7 PC3, and 0/5 VCaP cells were called as *PTEN* loss. 1/3 WBC cells are were called as *PTEN* loss. No cells analyzed in this group were observed to have a *PTEN* amplification. While we detected a reduction in normalized copy number for *PTEN* in the LNCaP cells, it did not reach statistical significance for deletion for most of the single cells analyzed. This is likely due to the multiploidy nature of this cell line, which may compress our sensitivity to identify heterozygous loss. In the 1 PC3 cells where *PTEN* loss was not observed, this was likely due to isolation of a normal WBC along with the PC3 cell of interest, leading to detection of the 2 copies of *PTEN* contributed by the contaminating WBC.

To address the possibility of detecting false CNVs due to the WGA process, we compared our NGS results to previously published Single Nucleotide Polymorphism (SNP) arrays on the LNCaP and PC3 cell lines [46]. We were able to recapitulate by NGS a subset of genes found to either be amplified or deleted by SNP array in both cell lines (S2 Table), indicating that our method can accurately reproduce known CNVs.

### Minimal Sequencing Depth Requirement

We estimated the minimal amount of reads required for reliable determination of single cell genomic instability and other alterations *in silico*. Our CNV analysis pipeline was performed on down sampled (50%, 10%, 5% and 1% reads) single cell data from LNCaP, PC3, and VCaP cells. We observed consistent genomic instability scores detected with >~350K reads (S1D Fig), and less reliable genomic instability scores were observed with lower coverage. Given this finding, we are using 350K reads as the minimum number of reads cutoff in our QC.

### Genomic Instability is Heterogeneous in Prostate Cancer CTCs

67 CTCs from 7 mCRPC cancer patients were sequenced, and the average number of CTCs sequenced per patient was 9.57 with a range of 2–17 CTCs per patient. Observed within this patient cohort is a wide range of CTC/mL counts, percentages of CK positive vs CK negative CTCs, and percentages of AR N-term positive and AR N-term negative CTCs, both within and between patients, reflecting the heterogeneous nature of the disease.

**Fig 5:**
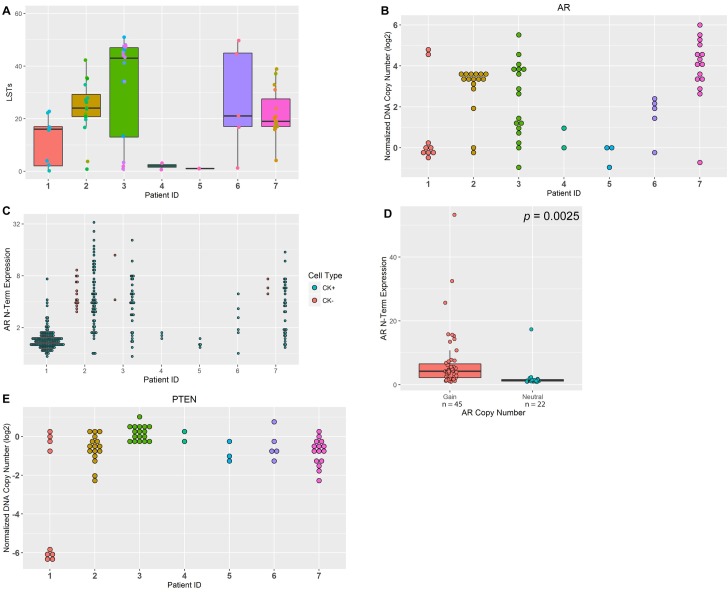
Genomic instability and CNVs in mCRPC patient CTCs. (A) Box-whisker plot of LST scores for patient CTCs. High LST scores were observed in 5/7 (71%) patients. (B) Dot plot of log2 normalized DNA copy number in *AR*. Amplification of the *AR* gene was observed in 5/7 (71%) patients. (C) Dot plot of AR N-Terminal protein expression status in each single CTC as detected by IF, 5/7 (71%) patients had amplified AR protein, where AR amplification is observed in both CK positive and CK negative CTCs. (D) Box-whisker plot of AR N-Terminal protein expression in AR copy number gain and copy number neutral CTCs. Higher AR protein expression was observed in the *AR* copy number gain group, *p =* 0.0025 by Student’s t-test. (E) Dot plot of log2 normalized DNA copy number in *PTEN*. Loss of *PTEN* was observed in 5/7 (71%) patients. In each figure, one dot represents a single CTC.

The distribution of LSTs was evaluated in patient CTCs. The majority of patients presented unstable CTC genomes except patients 4 and 5, which had few CTCs (2 and 3 CTCs, respectively) (Fig 5A, S3 Table). For patients with unstable genomes, heterogeneous LST scores were observed when analyzed at the single cell level, as summarized in S3 Table.

### *AR* Copy Number Concordance in Prostate Cancer CTCs

*AR* DNA amplification was detected in 5 out of 7 patients (patient IDs 1, 2, 3, 6, 7) (Fig 5B), where a wide range of *AR* amplification was observed at the inter- and intra-patient level. Consistent with this finding, expression of AR protein (AR N-term) was detected in the same 5 out of 7 patients (Fig 5C). Significantly higher AR protein expression was observed within individual CTCs harboring *AR* copy number gain compared to *AR* neutral CTCs (*p =* 0.0025) (Fig 5D), suggesting *AR* gene amplification directly correlates to AR protein expression.

### *PTEN* Copy Number Concordance in Prostate Cancer CTCs

*PTEN* loss was observed in 5 out of 7 patients (patient IDs 1, 2, 5, 6, 7) using CNV (Fig 5E, Table 3). To demonstrate concordance using an orthogonal method, single CTCs from the same patient cohort were analyzed for *PTEN* loss by FISH. Two patients have homozygous and one had hemizygous loss of *PTEN* confirmed by *PTEN* FISH, which correlated with NGS results (patient IDs 1, 6, and 2, respectively), whereas 2 patients were classified as non-deleted by both FISH and NGS (patient IDs 3 and 4) (Table 3). Representative FISH images from diploid and polyploid CTCs are shown in S2 Fig. Summaries of *PTEN* status as determined by FISH are shown in S3 Fig.

**Table 3:** *PTEN* status detected by CNV and FISH in patient CTCs.

Patient ID	1	2	3	4	5	6	7
***PTEN* status by CNV**	Loss	Loss	Non-Deleted	Non-Deleted	Loss	Loss	Loss
**Number evaluated**	5/9 Deleted; 4/9 Non-Deleted	3/16 Deleted; 13/16 Non-Deleted	17/17 Non-Deleted	2/2 Non-Deleted	1/3 Deleted; 2/3 Non-Deleted	1/5 Deleted; 4/5 Non-Deleted	5/15 Deleted; 10/15 Non-Deleted
***PTEN* status by FISH**	HO Loss	HE Loss	Non-Deleted	Non-Deleted	Non-Deleted	HO Loss	Non-Deleted
**Number evaluated**	5/68 HO Loss; 63/68 Non-Deleted	44/59 HE Loss; 15/59 Non-Deleted	27/27 Non-Deleted	5/5 Non-Deleted	1/19 HE Loss; 18/19 Non-Deleted	1/7 HO Loss; 6/7 Non-Deleted	44/44 Non-Deleted

## Discussion

Here we report the analytical and clinical feasibility of integrating single cell CNV analysis with the Epic Sciences CTC Platform. Focal CNV events and genomic instability, as measured by the number of LSTs, were reliably detected. The assay demonstrated intra-cell line reproducibility and accurately recapitulated known CNV events across the three well-characterized prostate cancer cell lines. Interestingly, VCaP had a similar number of LSTs compared to PC3. This suggests that LSTs may represent different mechanisms of genomic instability potentially driven by the loss of different tumor suppressor genes, such as *PTEN*, or impaired DNA repair pathways. Further clinical studies are planned to understand the clinical significance of this observation.

We have developed a post-sequencing data QC criterion comparing the ratio of residuals of LOESS fitting over total sequencing reads, which allows us to reliably filter out the samples not suitable for genome wide CNV analysis due to poor library complexity. *In silico* down sampling of the reads was performed to estimate the minimal number of reads required for detection of genomic instability scores. This analysis suggests that as few as 350K reads/cell (0.01x coverage) are required for reproducible assay performance.

We demonstrated the reproducibility of the assay by interrogating genomic changes such as *AR* amplification and *PTEN* loss in cell line cells, in addition to assessing the extent of genomic instability as a result of these changes. Both array comparative genomic hybridization (aCGH) based and NGS-based CNV analysis methods detect gains and losses relative to the ploidy level [46,47]. Because the ploidy levels may vary in tumor genomes, the log2 normalized copy number was calculated using most of the chromosomes as the baseline within each cell. Detection of gene gains or losses could be complicated or limited by abnormal ploidy. Comparison of *PTEN* FISH and sequencing based CNV results could be even further complicated because for FISH, *PTEN* deletion was determined by comparing *PTEN* copy number with chromosome 10 centromeres regardless of whole genome ploidy. In this study, we did the direct comparison of *PTEN* FISH results with sequencing CNV results, and some discordance should be accounted for due to the aforementioned limitations.

On the patient level, a strong correlation was observed between genomic *AR* amplification and AR protein expression. Five *AR* amplification patients had CTCs that express AR protein, while the two patients with normal *AR* copy numbers had CTCs that are negative for AR protein expression. Robinson *et al*. reported that 62.7% of AR aberrations and 71.3% of AR pathway aberrations were identified in 150 mCRPC patients [45]. This is consistent with recent study on a larger cohort that demonstrated the potential contribution of *AR* amplification to AR activation [48].

*PTEN* status was consistent in 5/7 patient samples between CNV and FISH with two discrepancies. Patients 5 and 7 were determined as having *PTEN* loss in CTCs by CNV analysis, but not by FISH. There were two potential reasons that may have contributed to the discrepancy: sampling error due to a small number of CTCs available for testing or low percentage of CTCs carrying *PTEN* loss, or false detection of gene loss in cells with multi-ploidy genomes. Patient 5 was determined to be *PTEN* non-deleted by FISH, with 1/19 CTCs harboring a hemizygous *PTEN* loss, where the sample did not reach the minimum cutoff of 3 CTCs for hemizygous loss by FISH, however by CNV analysis our criteria determined that 1/3 CTCs were deleted for *PTEN* resulting in a call of *PTEN* loss, suggesting low prevalence of *PTEN* loss in this patient. For this patient, the difference between FISH and NGS was not significant by Fisher’s exact test (*p* = 0.2597), suggesting that if we analyzed more CTCs by FISH, more *PTEN* hemizygous loss cells would be detected. Patient 7 was given an overall classification of *PTEN* loss by CNV with 5/15 cells scored as deleted, however 44/44 CTCs were classified as non-deleted by FISH, with potential polyploidy. The CTCs sequenced from this patient indicate that there is a high degree of polyploidy across most chromosomes, possibly contributing to false CNV calls.

The continual reduction of sequencing costs combined with the relatively low number of reads required to perform this assay enables the analysis of multiple individual CTCs from a single patient sample. Using a highly multiplexed system with a low number of reads per sample required to determine copy number changes, our single cell NGS analysis becomes more cost-effective than comparable methods, such as aCGH or SNP arrays. Additionally, our CNV methods are more sensitive, are less subject to noise, and require approximately 10-fold less starting material than array-based methods. With the multiplexed analysis by NGS, our system is capable of analyzing 96 samples simultaneously, increasing the throughput compared to array-based methods, which is typically limited to 8 samples per chip. Furthermore, these NGS methods allow for analysis of samples subjected to fixation, which frequently results in DNA of poor quality, where array-based methods would not be possible [49]. NGS also has an unlimited potential to detect CNVs in every genomic locus simultaneously, providing a clear advantage over other cytological methods, such as FISH, that that are limited by the number of probes that can be hybridized in a single reaction. Although it is feasible to use low pass whole genome sequencing to characterize genomic instability by LSTs, the detection of other genomic instability markers, such as single nucleotide variation (SNV), LOH and micro-satellite instability, still require allelic frequency information for their detection. Targeted resequencing methods from single cells compatible with the Epic Sciences platform are currently being developed.

While microarray and sequencing-based CNV assays have been available for years and are frequently performed from tumor biopsy samples, these assays are limited by the availability of sufficient amount of sample, tumor purity, biopsy sampling error and intra-tumor heterogeneity. The single CTC CNV assay described here enables evaluation of tumor heterogeneity, the presence of genomic driver alterations and genomic instability events that may potentially affect patient selection and therapeutic efficacy. Here we observed both inter- and intra-patient heterogeneity with respect to LSTs, *AR* amplification and *PTEN* loss across CTCs. A broad range of copy number changes in *AR* and *PTEN* were observed in most patients analyzed. The increased resolution of single cell CNV analysis allows for characterization of sub-clonal driver alterations, their clustering in sub-clonal populations (i.e., private or public alterations), and the weighted average of targetable pathways within a single patient. These tools will greatly aid drug development and stratification of patients for therapeutic combination strategies and clinical trials.

## Supporting Information

S1 Fig Assay reproducibility and minimal sequencing depth requirements. Circos plots of whole genome CNV profiles of each independent biological replicate from (A) LNCaP (*n* = 8), (B) PC3 (*n =* 7), and (C) VCaP (*n =* 5) cell lines demonstrate assay reproducibility. Each ring is the CNV profile from a single cell. (D) Down-sampling of reads from LNCaP, PC3, and VCaP cell lines from 3.5 x 10^4^ reads to 3.5 x 10^6^ reads establish the minimum reads requirement (350K) for detecting LSTs.
(TIF)

S2 Fig Representative FISH images. 2-color FISH images for patients using probes against *PTEN* (red) and CEP10 (green), with DAPI-stained nuclei (blue). Shown are example images of (A) a *PTEN* non-deleted diploid CTC with 2 CEP10 and 2 *PTEN* signals and (B) a *PTEN* non-deleted polyploid CTC with 4 CEP10 and 4 *PTEN* signals. The surrounding WBCs harbor diploid nuclei with 2 CEP10 and 2 *PTEN* signals.
(TIF)

S3 Fig FISH Scoring. (A-G) Scoring matrices for all 7 patients in this study for every CTC evaluated by *PTEN* FISH. Shown are the frequencies of CEP10 and PTEN in CTCs from every patient. The frequency of each PTEN signal was correlated to the CEP10 signal and is presented as a number of occurrences and percentage of CTCs. Each cell was scored as HE Loss, HO Loss, *PTEN* non-deleted, or *PTEN* gain. (H) Summaries of CTCs analyzed by *PTEN* FISH and *PTEN* status for all patients in the study.
(TIF)

S1 Table Correlation of Single Cell Sequencing to Pooled Cells. Correlation matrix comparing single cells with pools of 5 and 10 cells (n = 5 each) from each cell line. Colors indicate the degree of correlation where green is high correlation and red is low correlation. By concordance analysis, the average incidence of private CNV events for a single LNCaP cell is 12.7% (range: 9.3%-15.4%); for PC3, the average is 3.4% (range: 1.2%-7.7%); for VCaP, the average is 11.1% (range: 4.3%-16.4%).
(DOCX)

S2 Table Correlation of CNVs by NGS to SNP Arrays. (DOCX)

S3 Table Patient CTC Genomic Instability Scores. (DOCX)
