# Supplementary figures and images for: Chromosomal Instability Estimation Based on Next Generation Sequencing and Single Cell Genome Wide Copy Number Variation Analysis
Source: PLoS One. 2016 Nov 16;11(11):e0165089. doi: 10.1371/journal.pone.0165089 (PMC5112954; doi:10.1371/journal.pone.0165089)

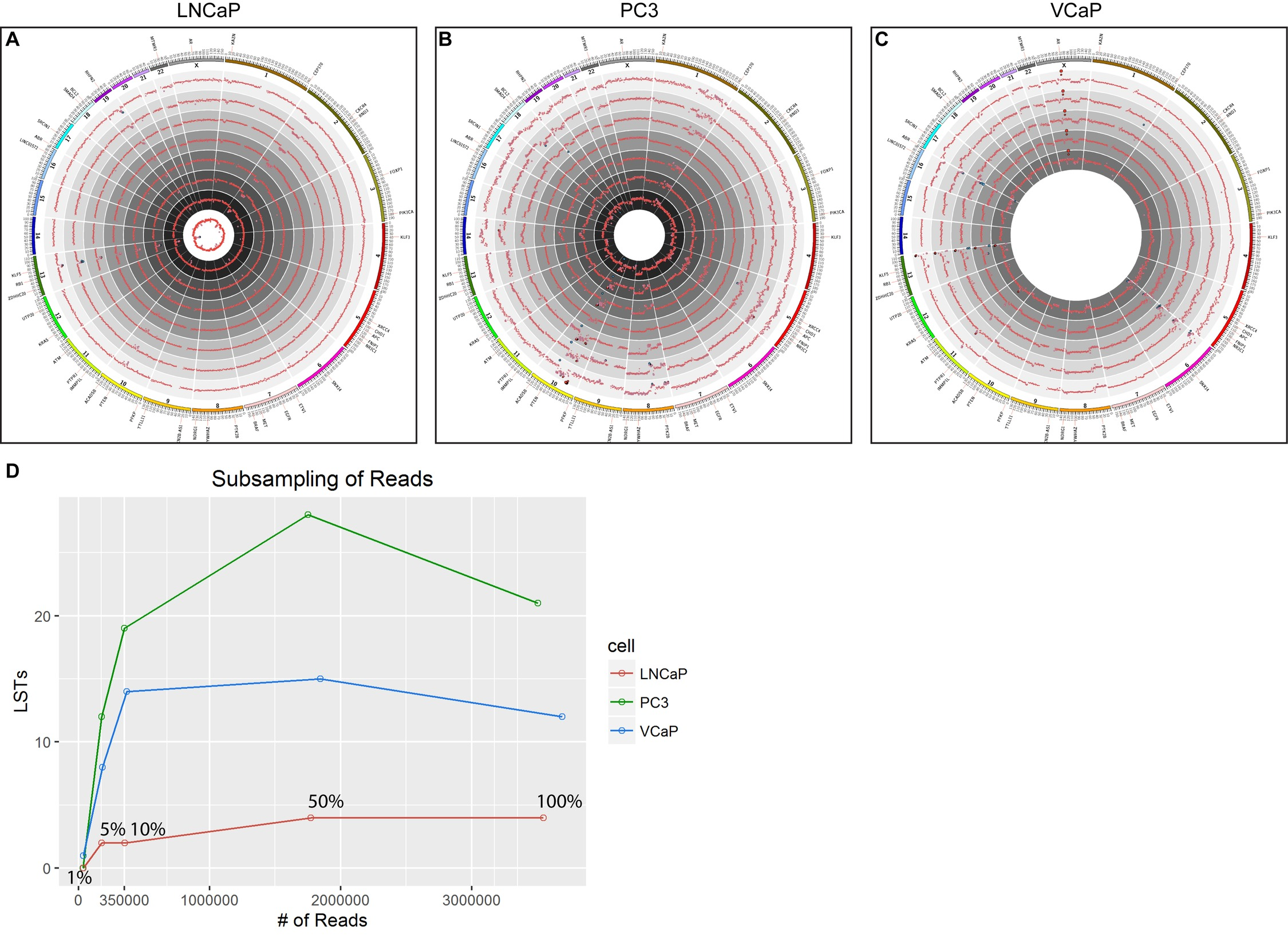

Supplement: S1 Fig — Circos plots of whole genome CNV profiles of each independent biological replicate from (A) LNCaP (n = 8), (B) PC3 (n = 7), and (C) VCaP (n = 5) cell lines demonstrate assay reproducibility. Each ring is the CNV profile from a single cell. (D) Down-sampling of reads from LNCaP, PC3, and VCaP cell lines from 3.5 x 10^4^ reads to 3.5 x 10^6^ reads establish the minimum reads requirement (350K) for detecting LSTs. (TIF) [file pone.0165089.s001.tif]

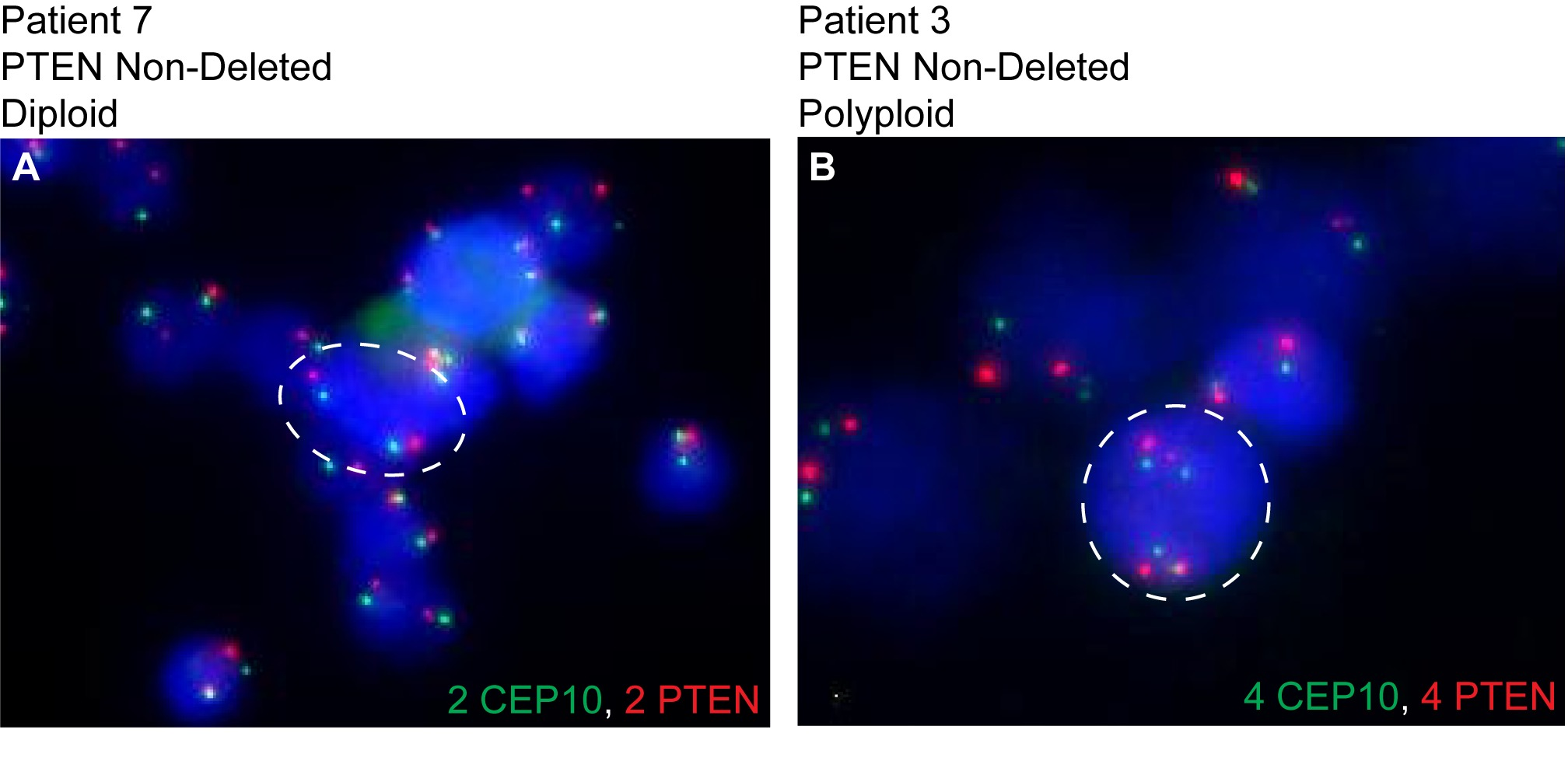

Supplement: S2 Fig — 2-color FISH images for patients using probes against PTEN (red) and CEP10 (green), with DAPI-stained nuclei (blue). Shown are example images of (A) a PTEN non-deleted diploid CTC with 2 CEP10 and 2 PTEN signals and (B) a PTEN non-deleted polyploid CTC with 4 CEP10 and 4 PTEN signals. The surrounding WBCs harbor diploid nuclei with 2 CEP10 and 2 PTEN signals. (TIF) [file pone.0165089.s002.tif]

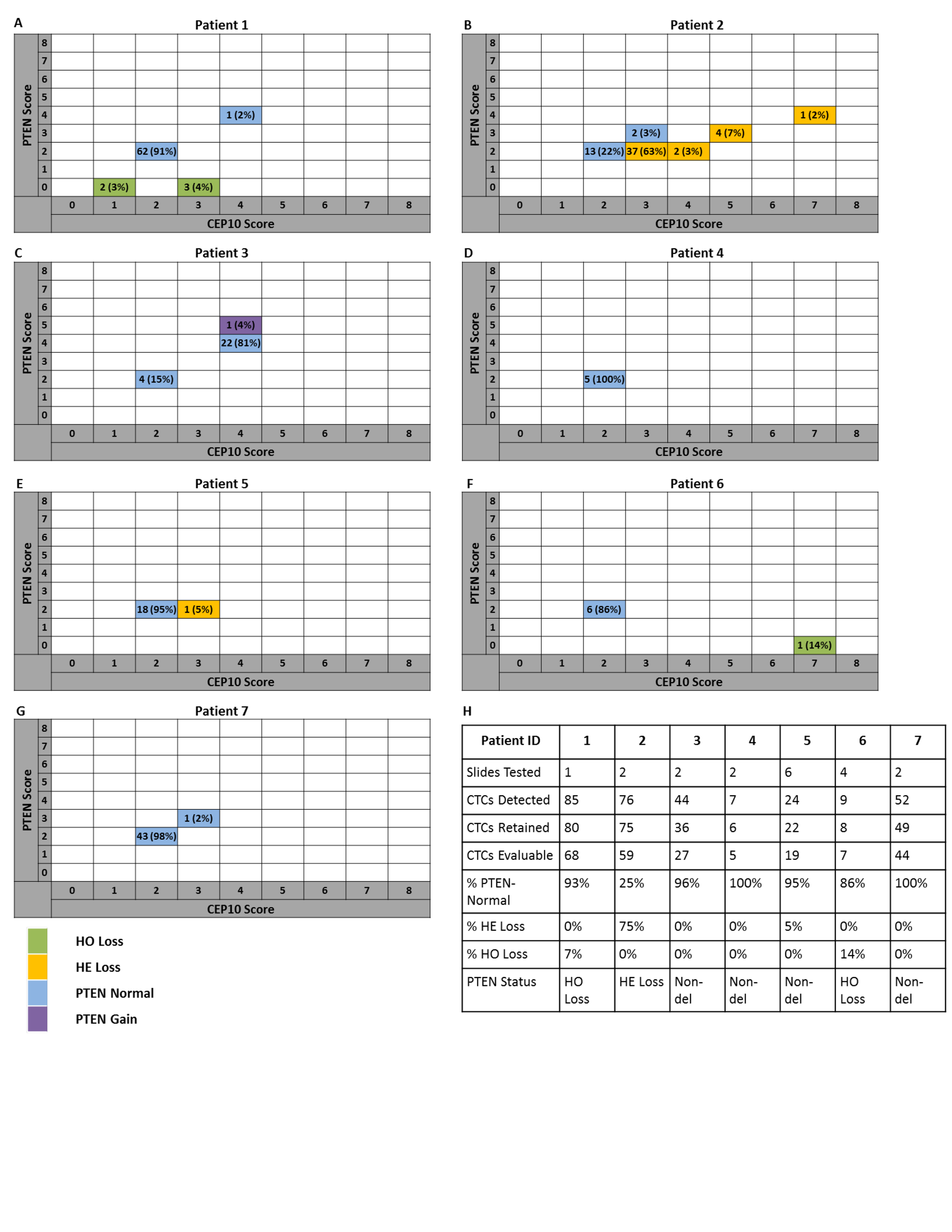

Supplement: S3 Fig — (A-G) Scoring matrices for all 7 patients in this study for every CTC evaluated by PTEN FISH. Shown are the frequencies of CEP10 and PTEN in CTCs from every patient. The frequency of each PTEN signal was correlated to the CEP10 signal and is presented as a number of occurrences and percentage of CTCs. Each cell was scored as HE Loss, HO Loss, PTEN non-deleted, or PTEN gain. (H) Summaries of CTCs analyzed by PTEN FISH and PTEN status for all patients in the study. (TIF) [file pone.0165089.s003.tif]
