# Supplementary material for: Chromosomal Instability Estimation Based on Next Generation Sequencing and Single Cell Genome Wide Copy Number Variation Analysis
Source: PLoS One. 2016 Nov 16;11(11):e0165089. doi: 10.1371/journal.pone.0165089 (PMC5112954; doi:10.1371/journal.pone.0165089)
Supplement: S1 Table — Correlation matrix comparing single cells with pools of 5 and 10 cells (n = 5 each) from each cell line. Colors indicate the degree of correlation where green is high correlation and red is low correlation. By concordance analysis, the average incidence of private CNV events for a single LNCaP cell is 12.7% (range: 9.3%-15.4%); for PC3, the average is 3.4% (range: 1.2%-7.7%); for VCaP, the average is 11.1% (range: 4.3%-16.4%). (DOCX) [file pone.0165089.s004.docx]

### S1 Table. Correlation of Single Cell Sequencing to Pooled Cells.

|  | | **LNCaP** | | | **PC3** | | | **VCaP** | | |
| --- | --- | --- | --- | --- | --- | --- | --- | --- | --- | --- |
|  |  | **1 cell** | **5 cells** | **10 cells** | **1 cell** | **5 cells** | **10 cells** | **1 cell** | **5 cells** | **10 cells** |
| **LNCaP** | **1 cell** | **0.92** |  |  |  |  |  |  |  |  |
|  | **5 cells** | **0.91** | **0.91** |  |  |  |  |  |  |  |
|  | **10 cells** | **0.88** | **0.90** | **0.90** |  |  |  |  |  |  |
| **PC3** | **1 cell** | **0.45** | **0.49** | **0.54** | **0.96** |  |  |  |  |  |
|  | **5 cells** | **0.41** | **0.49** | **0.52** | **0.96** | **0.98** |  |  |  |  |
|  | **10 cells** | **0.46** | **0.52** | **0.55** | **0.97** | **0.97** | **0.98** |  |  |  |
| **VCaP** | **1 cell** | **0.78** | **0.85** | **0.79** | **0.48** | **0.63** | **0.55** | **0.88** |  |  |
|  | **5 cells** | **0.78** | **0.86** | **0.82** | **0.46** | **0.62** | **0.53** | **0.88** | **0.94** |  |
|  | **10 cells** | **0.78** | **0.85** | **0.82** | **0.50** | **0.65** | **0.56** | **0.89** | **0.92** | **0.90** |
