# Supplementary material for: Chromosomal Instability Estimation Based on Next Generation Sequencing and Single Cell Genome Wide Copy Number Variation Analysis
Source: PLoS One. 2016 Nov 16;11(11):e0165089. doi: 10.1371/journal.pone.0165089 (PMC5112954; doi:10.1371/journal.pone.0165089)
Supplement: S2 Table — (DOCX) [file pone.0165089.s005.docx]

### S2 Table. Correlation of CNVs by NGS to SNP Arrays

| **Gene** | **Cell Line** | **SNP Array[**[**46**](#_ENREF_1)**]** | **NGS** |
| --- | --- | --- | --- |
| KCNK12 | LNCaP | Homozygous Deletion | 8/8 |
| MSH2 | LNCaP | Homozygous Deletion | 7/8 |
| CTNNA1 | PC3 | Homozygous Deletion | 6/7 |
| PTEN | PC3 | Homozygous Deletion | 6/7 |
| DNM3 | PC3 | Amplification | 6/7 |
| MYOC | PC3 | Amplification | 6/7 |
| NR5A2 | PC3 | Amplification | 6/7 |
| VAMP4 | PC3 | Amplification | 5/7 |
