# Supplementary material for: Chromosomal Instability Estimation Based on Next Generation Sequencing and Single Cell Genome Wide Copy Number Variation Analysis
Source: PLoS One. 2016 Nov 16;11(11):e0165089. doi: 10.1371/journal.pone.0165089 (PMC5112954; doi:10.1371/journal.pone.0165089)
Supplement: S3 Table — (DOCX) [file pone.0165089.s006.docx]

### S3 Table. Patient CTC Genomic Instability Scores.

| **Patient ID(CTCs sequenced)** | **LST** | | |
| --- | --- | --- | --- |
|  | **mean** | **Sd** | **Coefficient Variation*** |
| **1** (n=9) | 11.1 | 9.5 | 86% |
| **2** (n=16) | 23.8 | 10.7 | 45% |
| **3** (n=17) | 32.2 | 19.6 | 61% |
| **4** (n=2) | 2.0 | 1.4 | 71% |
| **5** (n=3) | 1.0 | 0.0 | 0% |
| **6** (n=5) | 26.8 | 20.4 | 76% |
| **7** (n=15) | 22.1 | 9.3 | 42% |

*Coefficient of variation is defined as the ratio of the standard deviation σ to the mean µ
